# Efficacy of new generation biosorbents for the sustainable treatment of antibiotic residues and antibiotic resistance genes from polluted waste effluent

**DOI:** 10.1016/j.imj.2024.100092

**Published:** 2024-02-19

**Authors:** Barkha Madhogaria, Sangeeta Banerjee, Atreyee Kundu, Prasanta Dhak

**Affiliations:** a Department of Microbiology, Techno India University, West Bengal, EM-4 Sector-V, Salt Lake City, Kolkata 700091, West Bengal, India; b Department of Chemistry, Techno India University, West Bengal, EM-4 Sector-V, Salt Lake City, Kolkata 700091, West Bengal, India

**Keywords:** Antibiotic pollutants, Remediation, Wastewater, Biosorption, Antibiotic resistance

## Abstract

Antimicrobials are frequently used in both humans and animals for the treatment of bacterially-generated illnesses. Antibiotic usage has increased for more than 40% from last 15 years globally per day in both human populations and farm animals leading to the large-scale discharge of antibiotic residues into wastewater. Most antibiotics end up in sewer systems, either directly from industry or healthcare systems, or indirectly from humans and animals after being partially metabolized or broken down following consumption. To prevent additional antibiotic compound pollution, which eventually impacts on the spread of antibiotic resistance, it is crucial to remove antibiotic residues from wastewater. Antibiotic accumulation and antibiotic resistance genes cannot be effectively and efficiently eliminated by conventional sewage treatment plants. Because of their high energy requirements and operating costs, many of the available technologies are not feasible. However, the biosorption method, which uses low-cost biomass as the biosorbent, is an alternative technique to potentially address these problems. An extensive literature survey focusing on developments in the field was conducted using English language electronic databases, such as PubMed, Google Scholar, Pubag, Google books, and ResearchGate, to understand the relative value of the available antibiotic removal methods. The predominant techniques for eliminating antibiotic residues from wastewater were categorized and defined by example. The approaches were contrasted, and the benefits and drawbacks were highlighted. Additionally, we included a few antibiotics whose removal from aquatic environments has been the subject of extensive research. Lastly, a few representative publications were identified that provide specific information on the removal rates attained by each technique. This review provides evidence that biosorption of antibiotic residues from biological waste using natural biosorbent materials is an affordable and effective technique for eliminating antibiotic residues from wastewater.

## Highlights

• One approach to combat increasing antibiotic resistance and the resulting rise in mortality rates worldwide is to remediate or biodegrade antibiotics contaminating wastewater.
• Removal of antibiotic residues from sewage water using conventional technologies has provided satisfactory results but the disadvantages cannot be ignored.
• This review suggests an alternative method, known as biosorption that can remove contaminants through adsorption of biological waste.
• Biosorbents are natural substances that are effective, easy-to-use, and inexpensive.
• Genetically modified plants and microorganisms should be investigated that can enhance the biosorption efficiency over a short time period.

## 1 Introduction

Antibiotics are used extensively in human and veterinary medicine. They have also been effective and conducive to growth in many industries, including animal and crop husbandry, aquaculture, and beekeeping [1], [2], [3], [4]. However, the presence of antibiotics in the environment has become an issue of increasing concern because of its impact on the emergence of antibiotic-resistant genes and the decreased efficacy of the antibiotic treatment of infectious diseases. Antibiotic residues are widely dispersed in soil and water as a result of antibiotic overuse, and sources of untreated water may contain high levels of antibiotics [5,6]. The evolution of resistance genes even at low levels is a major concern, with potential long-lasting detrimental effects on medical treatment. Particularly regarding regularly prescribed antibiotics, such as beta-lactams, sulfonamides, tetracycline, macrolides, fluoroquinolones, and cephalosporins. Antibiotic consumption increased by 91% between 1985 to 2021 [7,8]. Countries with the highest consumption of antibiotics are developing countries with large populations, such as India and China [9], [10], [11], [12]. In these two countries, the predominant antibiotics used are cephalosporins and tetracyclines [13]. The rising global population, the prevalence of newly emerging infectious diseases, and increased urbanization are all contributing factors to the rise in antibiotic consumption rates [14]. Tetracycline antibiotics are widely used in animal production worldwide [15,16]. Importantly, the body only processes or absorbs a small portion of the supplied human or animal antibiotic, with the majority of the drug instead being expelled in the urine and faeces. Some antibiotics with broad-spectrum activity can stay in the body for a long period of time before being excreted [17]. As a result, both the intact antibiotics that have been excreted and their metabolites eventually enter the environment. Another concern is that additional antibiotics are entering the environment via the direct disposal of unused or expired medications in drains, toilets, household garbage, and hospital solid waste [15]. In hospitals and in the community, antimicrobial resistance to pathogenic strains of bacteria has reached a high level and current efforts to develop novel therapeutics are insufficient to prevent a dramatic global increase in bacterial pathogens resistant to antibacterial agents. Despite the development of approximately 4000 new immunotherapies, only 30 to 40 novel antibacterials are currently in clinical trials and those that specifically target the World Health Organization's “Priority Pathogens” are derivatives of already established classes [18].

In addition to this therapeutic approach to combat increasing antibiotic resistance, we also need to remediate or biodegrade the antibiotics contaminating wastewater to control this pollutant and to reduce the mortality rates worldwide. Several studies, including laboratory and large-scale trials, have suggested that adsorption is the most effective method for removing antibiotics from wastewater [19]. A variety of factors, such as pH, ionic strength, temperature, and organic matter, significantly affect how efficiently antibiotic residues present in wastewater are absorbed [20]. Studies on tetracycline found high biosorption levels from wastewater and this was a result of its strong polarity and the presence of ionic groups [21]. Antibiotics can be biosorbed by various types of biosorbents via intermolecular forces namely, hydrogen bonds and van der Waal's forces, as well as by multiple interactions namely, cation exchange, electrostatic interactions, bon-bridges, co-ordination, and complexation [19,22]. This biosorption process is therefore an easy, rapid, and effective remediation method for the removal of antibiotics from wastewater.

In recent years, several reviews examined the fate and removal of antibiotics using biosorbents and provided information about the biosorption pathways for different antibiotic classes. However, in-depth analyses of the biosorption process for the removal of antibiotics are still lacking. The rapid increase in antibiotic resistance highlights the need to remediate or biodegrade antibiotics contaminating wastewater and different biosorbents have been employed based on the characteristics of specific antibiotics. The structures and functional groups of antibiotics may be the main factors affecting biosorption behaviour.

The fate and removal of antibiotics using biosorbents have been the subject of multiple reviews in recent years, and these reviews have also offered details on the processes and biosorption pathways for several antibiotic classes. However, in-depth research on the biosorption mechanism for antibiotic elimination is still lacking. This review article on the biosorption of antibiotics onto biosorbent materials offers new perspectives on antibiotic–biosorbent interactions. In this review, the biosorption mechanisms of antibiotics in the wastewater environment as well as the influences of environmental conditions on the biosorption behaviours of antibiotics in wastewater are thoroughly studied and explored.

### 1.1 Global antibiotic resistance status

**Fig. 1:**
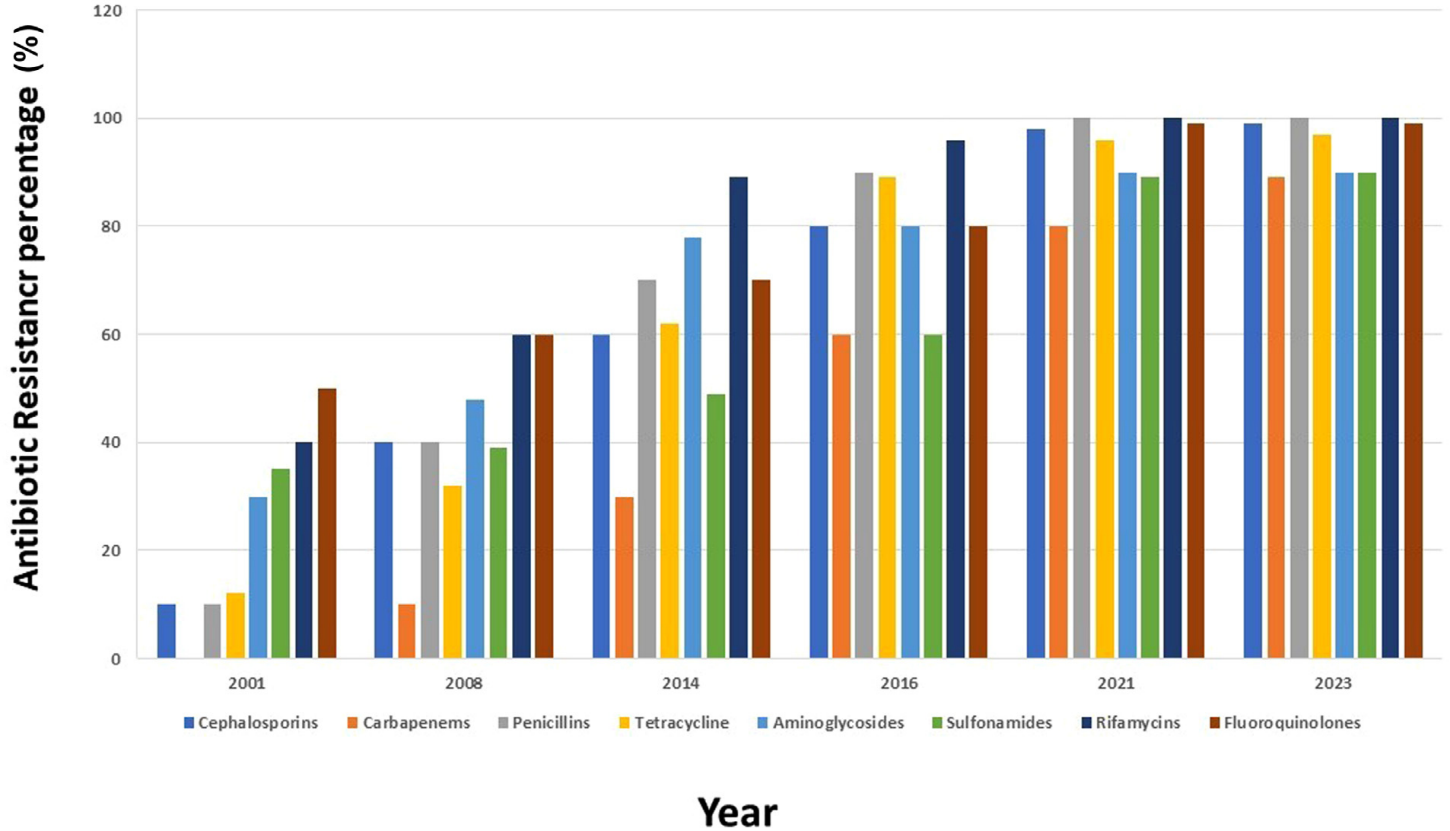
100% increase in resistance towards different classes of antibiotics over the years.

Antibiotics have transformed medicine since their discovery and have saved millions of lives [23]. However, antibiotic resistance has emerged as a result of the overuse of antibiotics, the lack of novel alternatives, low economic stature, and poor regulatory measures [24]. The uncontrolled spread of antibiotic-resistant bacteria has now become a global health issue [25] (Fig. 1).

New resistance mechanisms are continually emerging in bacteria and resistance genes are spread throughout bacterial populations as a result of selection pressure [26]. The spread of these genes affects the phenotypic characteristics of bacteria and makes therapeutic treatments ineffective, endangering human health. Antibiotic resistance genes (ARGs) are spread effectively through horizontal gene transfer [27,28]. Antibiotics are also found to contaminate wastewater. Conventional, mechanical, and biological sewage treatment methods cannot entirely remove all pollutants, and some remain within treated wastewater in surface water bodies. By consuming contaminated water, humans and animals are ultimately exposed to bacteria harbouring ARGs and antibiotic residues [26].

### 1.2 Antibiotic resistance mechanisms and dissemination

Antibiotic resistance is the characteristic of bacteria and other microbes to persist and grow in the presence of antibiotics that were previously lethal. Intrinsic resistance is a subcategory of antimicrobial resistance that refers to bacteria that are naturally resistant to a particular antibiotic because of their unique genetic composition. For example, some bacterial species harbour enzymes that can render antibiotics inactive (such as the AmpC lactamase from *Escherichia coli*). Acquired resistance is when a bacterium becomes resistant to an antibiotic via the acquisition of a resistance gene from a resistant bacterial strain. According to some studies, there are three main pathways of acquired resistance [29]. These include inactivation of antimicrobial agents through the addition of functional groups on binding sites or on the antimicrobial agent itself, decreased intracellular accumulation of microbial agents due to decreased influx or increased outflow, and modification of the target site for the antimicrobial agent [30].

The spread of antibiotic resistance among a bacterial population can occur via a number of processes, including transformation, transduction, and conjugation. Transformation is the process by which free or “naked” extracellular DNA passes through the cytoplasmic membrane into the cytoplasm [31]. Transduction involves the transmission of DNA between bacteria via a bacteriophage (i.e., a virus) that replicates within the bacterial cells. This process leads to recombination of the resistance sequence into bacterial DNA, conferring antibiotic resistance [32]. Conjugation involves the horizontal gene transfer of conjugative elements (such as plasmids and transposons) between donor and recipient cells [27].

Antibiotic therapy is challenged even further by the spread of multidrug-resistant (MDR) bacterial infections. MDR Gram-negative bacteria include *E. coli, Enterobacter aerogenes*, and *Klebsiella pneumoniae* (RL Rice, 2006). MDR can be acquired through a number of processes, such as membrane remodelling, drug inactivation, and drug modification (which can either increase drug efflux or decrease drug uptake). All possess efflux pumps, which, as a result of AcrABTolC overexpression, affect the minimum inhibitory concentrations (MICs) of a number of antimicrobial drugs. In a study by A. Abushaheen et al., in 2020 fluoroquinolone resistance was reported in all MDR isolates analysed, indicating significant selective pressure to develop quinolone resistance in bacteria [30].

## 2 Types of antibiotic residues, their effects, and their presence in wastewater reservoirs

Compounds or metabolites of antibiotics can accumulate in the cells and tissues of humans, possibly originating from the consumption of contaminated water or animal products, such as milk, eggs, and meat [33], [34], [35]. The accumulation of antibiotic residues may lead to severe pathological outcomes, such as autoimmune diseases, reproductive disorders, bone marrow toxicity, severe allergies, mutations, hepatotoxicity, immune pathological effects, and teratogenicity effects (i.e., toxic effects on an embryo or foetus during the critical phase of gestation) [36,37]. Various types of cancer, including pancreatic cancer, have also been associated with the accumulation of antibiotic residues [38]. Humans are the ultimate consumers of antibiotic residues present in the food chain [39,40]. These antibiotic residues are expressed in part by weight, such as mg/kg (ppm), ppb, or mg/kg (ppt) [33]. The maximum residue level/limit (MRL), also known as the tolerance level, is the maximum permissible level or concentration of an antibiotic used or present in feed, food, and wastewater that is referred to as safe for consumption by humans and animals [41,42]. The MRL of detection of an antibiotic residue in food or wastewater, based on toxicological NOEL (No-Observable-Effect-Level) and food factor values, is determined as follows:

MRL = ADI (acceptable daily intake for human) × with an average consumer body weight (60 kg) / food factor or water factor × 0.5 kg food/water [43]

MRL of amoxicillin in food = 0.004 ppm

MRL of ceftriaxone = 0.1 ppm

Below, is a description of the major reservoirs of wastewater potentially contaminated with antibiotic residues, the types of antibiotic residues involved, and the major sources of environmental discharge of antibiotics.

### 2.1 Major reservoirs

The major types of wastewater contaminated with antibiotic residues includes pharmaceutical, municipal, and sewage water from hospitals [44], [45], [46]. Wastewater samples are collected and analysed for antibiotic residue remediation using high-performance liquid chromatography (HPLC). In addition, Enzyme-linked Immunoassay (ELISA), liquid chromatography, gas chromatography, and paper chromatography are techniques that are frequently used for the detection and analysis of antibiotic residues [47,48].

#### 2.1.1 Pharmaceutical industry

The pharmaceutical industry is the major contributor to the release of antibiotics into the ecosystem [49]. India is a primary producer of pharmaceutical products, including antibiotics [50,51]. In Europe, various classes of antibiotics, such as macrolides, quinolones, sulfonamides, meropenem, imipenem, ciprofloxacin, and oxytetracycline, have been detected in the wastewater released from the pharmaceutical industry [52]. Pharmaceutical wastewater also contains a high concentration of veterinary antibiotics, which are used to enhance the growth and productivity of livestock and suppress or prevent infections [50,51,53,54].

#### 2.1.2 Hospitals

Hospital sewage is reported to be 15–20 times more hazardous than standard municipal sewage water [1,55]. Hospital wastewater includes effluents from laboratories, operation theatres, the radiology department, transfusion centres, wards, outpatient departments, medical shops, and hospital toilets [56]. Every year, hospitals discharge approximately 2,350 tons of used or unused pharmaceuticals as waste [57]. In fact, in most cases, antibiotics are not completely metabolized by patients and are instead excreted [57,58]. It has been reported that antibiotics released from hospitals are resistant to the processes undertaken at wastewater treatment plants [55]. Large quantities of fluoroquinolones and tetracycline have been detected in hospital wastewater, whereas beta-lactam antibiotics are less abundant because they are quickly decomposed by microorganisms present in the wastewater.

#### 2.1.3 Municipal

The major pathways through which household pharmaceuticals enter municipal wastewater are human excreta, wash-off, and household waste [59]. In a study in India, 56% of household waste was reportedly released through household drains [60]. Amoxicillin, tetracycline, and dicloxacillin have been reported in the drinking water in Japan [61]. In many countries, antibiotics such as sulfamethoxazole, trimethoprim, norfloxacin, and metronidazole are present in the wastewater released from households [62]. Whereas, in India, the predominant contaminating antibiotics are ciprofloxacin, levofloxacin, and norfloxacin, present in approximately 78%–90% of municipal wastewater [63,64].

### 2.2 Harmful effects of antibiotic residues on the environment, and on human and animal health

Hazardous waste, which is considered an emerging contaminant in sewage treatment plants, can be categorized according to various functional classifications as either P-listed, U-listed, D-listed, F-listed, or K-listed pollutants [65,66]. Antibiotic residues are classified as either F-listed or K-listed pollutants. An F-listed wastewater pollutant originates from a pharmaceutical, hospital, or municipal source, whereas a K-listed wastewater pollutant originates from a veterinary source [10]. Antibiotic residues can be passed to humans via several routes, including the consumption of contaminated plant material. Unlike animals, plants have no solid or liquid excretory system [65]. Chronic exposure to antibiotic contaminants mainly effects the human gut microbiome by decreasing immune development, pathogen colonization susceptibility, and food metabolism [67]. It has been reported that high levels of consumption or use of antibiotic-contaminated water or food can cause kidney blockage, autoimmune disease, and liver cancer [68]. The presence of tetracycline, beta-lactam, and enrofloxacin residues in raw cow's milk can induce liver and pancreatic damage in humans [69].

Antibiotics have potential genotoxic characteristics [70]. This has been demonstrated through the use of various animal models and microbiological experiments. The SOS chromo test on *E. coli* and the Ames test on *Salmonella* species are just two of the assays that have been developed to investigate the toxicity effects of antibiotics and their residues [71]. To assess the genotoxicity of antibiotics, higher plants have also been used as models [72]. For example, growth suppression was demonstrated in *Lemna minor* and *Scenedesmus vacuolatus* in response to florfenicol [73]. Human stem cell proliferation has been demonstrated to be delayed by chloramphenicol and rifampicin [74]. Ceftriaxone and doxycycline have cytotoxic and genotoxic effects on human peripheral blood lymphocytes. In animal models, penicillin has been reported to cause disruption of lipid metabolism [75]. It has been reported that 30 µg/mL to 200 µg/mL of fluoroquinolone, trimethoprim, sulfonamide, and tetracycline contaminated effluent discharge from pharmaceutical industries is attributed to multiple types of abnormalities in zebrafish embryos [50]. This implies that over the long term, the consumption of antibiotic residue-contaminated water causes significant toxicity to living organisms and risks the further development and dispersion of antibiotic resistance. Another previous study investigated the neurotoxicity of 100 µg/L of clarithromycin, 10 µg/L and 100 µg/L of chlortetracycline, and 10 µg/L and 100 µg/L of roxithromycin on *Danio rerio* (zebrafish) larvae, and found increased levels of cell apoptosis in brain sections along with changes in synaptogenesis, neurotransmission, mitochondrial stress response pathways, and the endocrine system, which induced neural damage [76]. Another study reported the impact of 100 µg/L of doxycycline, oxytetracycline, and florfenicol on the levels and composition of gut enzymes in *Danio rerio*, and reported reduced mucus secretion, decreased expression of genes encoding triglyceride, pyruvate, acid phosphatase, and total cholesterol, decreased levels of *Fusobacteria*, and increased levels of *Proteobacteria* [77]. This implies that subchronic exposure to antibiotic residues can induce dysbiosis and dysfunction of the gut microbiota of all life forms [50]. It has been demonstrated that long-term exposure to clindamycin administration induced accelerated levels of human intestinal clindamycin resistance genes in human DNA genetic material extracted from faeces [78].

## 3 Wastewater remediation of antibiotic residues using different techniques

As ARGs continue to spread rapidly, remediation techniques for the eradication of antibiotic residues have become an area to interest. Below, techniques employed for the remediation of antibiotic residues from wastewater are described, along with an explanation of the drawbacks that have limited their efficiency (also see Table 1).

**Table 1:** Advantages and disadvantages of different technologies used for the removal of antibiotic residues from waste water systems.

Antibiotic residue removal Processes	Advantages	Disadvantages
Floculation-coagulation	Detention time of waste water for the treatment is very less for this method. Additionally, multiple pollutants can be targeted by a single system attain many objectives	Chemical addition for the coagulation produces sludge having toxic compounds that need to be removed and treated after the process
Advanced oxidation processes Photolysis Photocatalysis Ozonation Fenton	Bromated products after the treatment is not formed A drinking water treatment plant can have this setting Organic matter degradation happens very efficiently Rapid degradation and meneralization of organic products	Turbidity can affect the light needed for the treatment Not cost effective It requires high cost Unknown by products are produced which needs to be analysed It's good for laboratory scale sludge is produced having large amount of ferrous and anions
Antibiotic in waste water	Biosorbent used	Efficacy and other factors
Tetracycline	Shrimp shell waste composed of chitin	*Q*_max_= 229.98 mg/g Removal efficiency = 96.79% Hydrogen and pi bond formation Time = 36 h Temp.= 55˚C pH = 3.3
Dicloxacillin	Tannin extracted from Terminalia cotappa leaves	*Q*_max_= 17.28 mg/g Removal efficiency = 94% Hydrogen and Vanderwaal formation Time = 24 h Temp.= 55˚C pH = 6.0
Biological Biodegradation	Low-cost and helps degrading pollutant	Microbes which helps in degradation can slow down the process due to factors like large amount of sewage its flow rate etc. which affects the treatment efficiency
Anaerobic treatment	Efficient in removing organic compounds with high strength	They can also remove nonorganic substances also which can are not pollutants
Aerobic treatment	Good for odour removal along with the treatment	Not very economical needs high maintenance and operating cost
Membrane filtration	Effluent quality is increased and can be used as pre-treatment very effectively	Fouling of membrane, installation cost is high, diverse types of membranes are needed

### 3.1 Dielectric barrier discharge method

The main disadvantage of this approach is the need for energy in the form of electric current, which is inappropriate for actual wastewater treatment [79]. This process is more expensive than other remediation methods. This method is also unable to remediate antibiotic residues in higher amounts along with ARGs and antibiotic-resistant bacteria [80]. Furthermore, with this technique, there is a high chance that secondary pollutants may be released as a by-product into the water, which is more harmful to the ecosystem [81,82]. The identification and determination of degradation pathways have not yet been studied, but are important to understand to achieve efficient remediation of antibiotic residues from wastewater.

### 3.2 Chlorination

Chlorination is one of the chemical methods for the removal of antibiotic residues from water [83]. Less research has been carried out on this type of remediation method to date. The process of chlorination involves complete disruption of the cell membrane, and the coagulation of enzymes and genetic material in the bacterial cell [51,83]. However, only a 25%–30% reduction in erythromycin and tetracycline residues was successfully remediated by this process [84]. The major drawbacks of exploiting chlorine-based decontaminators include the handling exposure risk and the production of pollutants in the form of secondary by-products [85,86]. To achieve significant remediation of antibiotic residues by this method, an adequate amount of free chlorine ions and a sufficient reaction time are required [87]. Another important drawback of this method is that chlorination of wastewater for the removal of antibiotic residues did not effectively inactivate or degrade antibiotic-resistant bacterial cells or ARGs [83,86,87]. This method was also unable to remediate toxic antibiotic residues from the wastewater completely. Therefore, plasmid-borne ARGs can potentially be transferred to other bacteria even after the chemical disinfection process [88]. To address this, more advanced treatment processes are needed for the effective and economically viable remediation of antibiotic residues along with ARGs and antibiotic-resistant bacteria from hospitals, municipal sources, and industrial wastewater.

### 3.3 Flocculation-coagulation

The coagulation-flocculation method (C-F) can be used at different stages throughout the water treatment process. This technique removes solutes from water and involves the addition of chemicals to the water to destabilize colloidal particles, followed by the aggregation of particles through flocculation and sedimentation. The maximum rates of removal of antibiotics from wastewater accomplished by this physicochemical process were 46%, 42%, and 23% for the antibiotics ibuprofen (IBP), naproxen (NPX), and diclofenac (DCF), respectively. Pharmaceuticals and personal care products (PPCP) such as, insect repellent, anti-inflammatory products compositions were unaffected by physicochemical treatment [89]. However, appreciable improvement in antibiotic levels was detected when aluminium or iron salt was used as a coagulant, so evidence for the effectiveness of this antibiotic removal technique is lacking. One drawback of this technique is the use of chemicals [88].

### 3.4 Advanced oxidation processes (AOPs)

AOPs are based on the generation of hydroxide ion (•OH), which is a by-product of the reaction between H_2_O_2_, O_3_, photocatalysis, or oxidants, mediated by sunlight or ultraviolet radiation. The equation, OH + R˗H → H_2_O + •R illustrates how hydroxyl radicals attack an organic compound and acquire a hydrogen atom (R˗H) to form an organic radical (•R). Numerous products are formed via chemical reactions that are mediated by this free radical.

Two categories of AOPs exist: (1) non-photochemical AOPs, such as Fenton, O_3_/H_2_O_2_, and O_3_. (2) Photochemical AOPs this includes methods in which to generate hydroxyl radical Ultraviolet light (UV light), H_2_O_2_, O_3_ and/or Fe^+2^ is being used [90]. The photochemical AOPs methods use UV light along with to generate reactive hydroxyl radical. Ozonation is involved in such processes. A pH >8 is required to attain high removal efficiency with ozone treatment, as this causes the ozone to rapidly break down into hydroxyl free radicals. Antibiotics and all other organic components should be oxidized at a pH of 8–10. The simplified reaction mechanism for ozone at a high pH is as follows: 3 O_3_ + H_2_O → 2 •OH + 4 O_2_. Fenton and photo Fenton reactions are examples of the photochemical group.

One simple technique for eliminating organic compounds, including antibiotics, from wastewater is the Fenton reaction. The advantage of adding UV-visible light is that it can significantly accelerate the dissolution of organic impurities added to the reaction. The following equations describe the mechanism involved: Fe^2+^ + H_2_O_2_ → Fe^3+^ + OH^‒^ + •OH Fe^3+^ + H_2_O → Fe^2+^ +H^+^ + •OH H_2_O_2_ + hv → 2 •OH [91].

In acidic environments, direct oxidation predominates in the ozonation reaction, which has a limited capacity to eliminate contaminants.

### 3.5 Heterogeneous photocatalysis

One method that is frequently used to treat water-based pollutants is heterogeneous photocatalysis, which involves acceleration of the photosynthetic reaction when a catalyst is present. Among its drawbacks is the need for high catalyst doses, which limits the efficiency of the process. Furthermore, using this method, may make it more difficult to separate and recycle an expensive photocatalyst, such as TiO_2_ [89]. Even after the antibiotic was treated four times over a duration sufficient for its degradation, 100% of the organic carbon remained during the electrochemical treatment. Whereas, the natural matrix, which was mineral water, did not considerably impede the removal of pollutants for any of the processes. However, the presence of glucose in water had a significant impact on the degradation of cephalosporin antibiotic that is cephalexin (CLX) by TiO_2_ photocatalysis [92]. Despite their excellent performance in the degradation of antibiotics, the primary issue with AOPs, which can produce a large number of oxidation intermediates and products, is their limited mineralization capacity [93,94]. The lasting or imperceptible toxicity of these degradation products is an issue of concern [95]. By employing the electrochemical method, the photo Fenton system, and the TiO_2_ photocatalysis process, cloxacillin was broken down by hydroxyl radicals, adsorbed •OH, and oxidation at the catalyst surface, respectively. While the antibiotic was effectively eliminated by all three oxidation processes, only photocatalysis using TiO_2_ demonstrated a significant level of total organic carbon removal (approximately 45%). Nevertheless, every treatment produced by-products that altered the penicillinic nucleus, which is the portion of the compound that has antimicrobial activity [92]. However, electrochemical oxidation has not been applied widely because of the high cost of electrode materials. Furthermore, slow mass transfer in the electrochemical oxidation reactor leads to low flow efficiency and high energy consumption when the wastewater has low conductivity. Low-cost anode materials with strong stability and catalytic activity are therefore required.

### 3.6 Membrane filtration

Four pressure-driven membrane processes exist that allow for separation in the liquid phase, namely microfiltration, ultrafiltration, nanofiltration, and reverse osmosis. Ultrafiltration was found to confer 80% antibiotic retention on a PVC membrane [96]. For nanofiltration, a study carried out using a hydrophilic and antifoulic Zwitterionic polyamide membrane exhibiting 96.5% antibiotic retention [95]. Membrane technology simply changes the state of antibiotics. In addition, dirt particles that accumulate in the pores and on the membrane surface weaken the membrane and make it more prone to cracking. As a result, the membrane filtration process sometimes decreases water flow through the membrane leading to less removal [97]. In an effort to increase the efficiency of wastewater treatment to remove antibiotic residues, hybrid systems have recently gained attention. Many types of hybrid system exist, one of which involves the incorporation of membrane technology with photocatalysis, termed a photocatalytic membrane reactor. The photocatalyst can be suspended in the wastewater or can be present on the membrane [98]. The placement can also differ, it can be submerged in the slurry with the photocatalyst or it can be pressurized on both sides by a pressurized circulation photocatalyst. To prolong membrane life and improve abrasion resistance and chemical stability, ceramic materials were considered. Oxytetracycline removal was studied using this hybrid system, where TiO_2_ was used as the catalyst and it was suspended in the wastewater, and the results confirmed complete removal of oxytetracycline with 47% mineralization. TiO_2_ has gained immense popularity as a result of its photocatalytic degradation properties [99].

### 3.7 Biological treatment

This method is conducted with the help of microorganisms. Both aerobic and anaerobic microorganisms can mediate biodegradation Aerobic biodegradation produces carbon dioxide and anaerobic degradation produces methane. Thus, biological treatment of antibiotics can be conducted in an aerobic, anaerobic, or hybrid manner. In this method, the biodegradability of the antibiotic is an important factor that is measured by a closed bottle test [99,100]. The following are some of the modern anaerobic technologies developed for the disposal of pharmaceutical waste: anaerobic membrane bioreactor (AnMBR), anaerobic digestion, anaerobic filter, anaerobic bio-entrapment membrane bioreactor (AnBEMR), up-flowing anaerobic sludge blanket (UASB), and anaerobic sequencing batch reactor (AnSBR) [99]. Regarding performance, the AnBEMR conferred 15% more methane production and 5%–10% greater removal of the chemical oxygen demand (COD) over AnMBR. AnBEMR also exhibited less production of extra polymeric substances, as well as soluble microbial products [101]. AnSBR is a five-step process carried out in a single reactor in the absence of light. In one study, the removal of erythromycin from wastewater using AnSBR was shown to take a long period of time. In an experiment carried out in the presence of white rot fungi, erythromycin and tetracycline were removed from biosolids using AnSBR. In another study using fungi, AnSBR was conducted to remove sulfamethoxale and the treatment efficiency was 40 mg/L, although the efficiency of the process was affected by the concentration of antibiotic [102,103]. UASB along with anaerobic treatment can also be used in the pretreatment of wastewater, and in one study, this technique removed 19%–33% of 6-aminopenicillanic acid (6-APA) and 13%–47% of amoxicillin [104]. The UASB method also showed a 95% removal rate of antibiotics when used as a pretreatment option [103]. Furthermore, with aerobic treatment, the biodegradation of sulfamethoxazole over 5 days led to 100% removal, and an equivalent tetracycline removal rate of 86.4%. The drawback of this method is that it can be used in diluted wastewater only, thus increasing the capital cost but recent developments is been done to overcome this problem especially in municipal sewage treatment plant in Brazil. In hybrid systems incorporating both the aerobic and anaerobic methods, there are two types of reactors: a sequencing batch reactor that includes more than one aeration tank sewage entrance, and a membrane bioreactor that is a hybrid of the membrane separation and biological methods. The membrane bioreactor showed 94.7% COD removal compared with the UASB process alone that conferred 41.3% removal efficiency. Another hybrid technology incorporating UASB a biological contact oxidation tank (BCOT), a novel micro-aerobic hydrolysis acidification reactor (NHAR), and cyclic activated sludge (CASS) showed COD treatment of up to 97% [99]. Sulfonamide antibiotics cannot be fully extracted using conventional activated sludge treatment techniques. However, microbial metabolism may slow down as a result of exposure to antibiotic wastewater, which could impair the effectiveness of biological wastewater treatment.

### 3.8 Adsorption

Although their application to the removal of antibiotics has only been documented for approximately 30 compounds to date, adsorption processes are widely used to remove organic contaminants from contaminated streams onto adsorbent surfaces [105,106]. The type of adsorbent, the characteristics of the adsorbate, and the composition of the wastewater all have a significant impact on the effectiveness of adsorption processes [107]. The characteristics of the adsorbent, such as the specific surface area, porosity (macro or micro porosity), pore diameter, and functional groups, are directly related to the adsorption efficiency [108]. A number of adsorption materials, such as ion exchange materials, natural clay materials bentonite, activated carbons, and carbon nanotubes—particularly multi-walled carbon nanotubes—are being investigated for the removal of antibiotics. Other adsorbents, such as kaolinite, MgO particles, MgO nanoparticles, ZnO-MgO nano composites, and hollow silica nanospheres, have also been considered as adsorption material [109]. In conclusion, adsorption is a successful technique that extracts antibiotics from contaminated water with an efficiency ranging from 90% to 100%. The main drawbacks of both activated carbons and carbon nanotube devices are the high material costs and the possible high regeneration costs. Adsorption kinetics frequently seemed to follow second order, but adsorption equilibrium was well-modelled by either Langmuir or Freundlich isotherms. Antibiotic adsorption most likely involves hydrogen bonding, pour filling, hydrophobic interactions, electrostatic interactions, and pi-pi (Electron-Donor-Acceptor) EDA interactions. There is growing interest in activated carbon adsorption technology because of its efficiency, large surface area, ease of use, and lower cost. A study reported the use of a composite of metal-organic framework and a polymer to perform sulfonamide adsorption with promising results [110]. The metal-organic framework polymer composite adsorbent was found to have fully adsorbed the sulfonamide. However, because of the strong Lewis acid–base interaction, the adsorbent was challenging to desorb. The drawbacks of this technique include the high energy requirement, ineffective disposal, the creation of hazardous by-products, and an excessive amount of secondary sludge that is detrimental to aquatic and human health.

## 4 Biosorption technique

Among the above-mentioned conventional methods for the removal of antibiotic residues from wastewater, the biosorption technique using appropriate biosorbents is a potentially promising and efficient method. This process has many advantages over conventional methods, such as its simplicity, cost-effectiveness, and ability to operate over a large surface area [83,111]. Recently, scientists have focused on the efficient and cost-effective biosorbents originating from biological sources. For example, several microorganisms, plants, agricultural waste, and fruit peel have been studied as potential biosorbents with binding capacities to different types of antibiotic residues present in wastewater. Among them, bioactivated carbon produced from several types of materials is extensively used and considered the most common and efficient biosorbent for the remediation of antibiotic residues [112].

The phenomenon of biosorption is based on liquid–solid intermolecular affinity forces [19]. In this context, antibiotic residues are known as biosorbates, while the antibiotic residues that are retained are known as biosorbents (biologically-derived). The binding of antibiotic residues on the surface of biosorbents is mediated by two major types of bonds, namely covalent bonds, which are considered strong bonds, and van der Waals forces, which are considered weak bonds [113]. Often, electrostatic attraction is also involved in this process. The biosorbents involved in this process have a porous surface structure [114]. In short, the biosorption process can be either physical or chemical depending on the interactions between the biosorbent and the biosorbate. For the biosorption of antibiotic residues from sewage water, bioactivated carbon is mainly utilized due to its static charge and hydrophobic nature [115,116]. A large surface area and porosity are two factors that contribute to effective biosorption. Depending on their origin, biosorbents are considered to be natural, synthetic, or biologically-derived. Natural biosorbents are those extracted from natural materials such as clay, charcoal, zeolites, ores, and clay minerals [114]. Synthetic biosorbents are those obtained from agricultural, industrial, domestic, plant or fruit waste [117]. Biologically-derived sorbents (biosorbents) are extracted from fungi, algae, bacteria, medicinal plants, and polyphenols [114]. Biosorption is a reversible process in which toxins adhere to the surface of a biosorbent [118]. Different biosorbents have unique properties such as porosity, pore size and shape, type and degree of ionic charge, type and number of functional groups, and the nature of the biosorption surface [40]. This type of biosorbent is relatively cheap, environmentally-friendly, and abundant in nature. In this context, the amount of sorption expresses the threshold amount of antibiotic residues adsorbed onto the surface of the biosorbent under the influence of factors such as pH, ionic charge, ionic strength, initial biosorption dose, mixing speed, surface area, structure, size, container, temperature, antibiotic residue, and functional groups [113]. Another important factor determining the sorption capacity is the type of interaction between the biosorbent and the biosorbate. For bio-waste compounds, the biosorbent biomass can be easily recycled through a desorption process, where these biosorbents completely desorb antibiotic residues without degrading the chemical or physical properties of the biosorbent [90].

A number of research studies have discussed the use of continuous fixed bed biosorption technology for the biosorption of antibiotic residues. This technique typically uses fixed-bed reactors and is mainly used in large-scale wastewater treatment [119]. This fixed bed reactor is simple and has high removal efficiency [92]. In this process, a biosorbent is immersed and packed into a column, and wastewater containing a certain concentration of antibiotic residues, known as the liquor, is passed through it; this is the basis for the design of a full-scale fixed-bed biosorption process. This process involves regeneration, for which there are numerous potential methods, including vapour desorption, thermal fluctuation, pH change, electrochemical technology, ultrasonic technology, and the use of desorption agents such as HCl, H_2_SO_4_, HNO_3_, NaOH, CaCl_2_, and ethylenediaminetetraacetic acid (EDTA) [56]. Not all of these techniques work with all types of biosorbents, and the choice of approach should take into account the biosorbent type, the wastewater conditions and quality, and the cost of regeneration. The absorption of biosorbents by used biosorbents allows them to be recycled in subsequent biosorption cycles, which reduces the need for new biosorbents. Moreover, dried, powdered forms of biosorbents are prepared at micro- or nano-scale. For designing eco-friendly nano-sized biosorbents, the green synthesis route is preferable as no toxic chemicals are used. The biosorption efficiency of a biosorbent for antibiotic eradication can be calculated by the following equation [118]:

(1) $$Q_{e}=\left(C_{o}-C_{e}\right)V/W,$$

where *Q*_e_ = biosorption efficacy

*C*_o_ = initial concentration of the antibiotic residual solution (mg/L)

*C*_e_ = equilibrium concentration of the antibiotic residual solution (mg/L)

*V* = volume of the antibiotic residual solution (L)

*W* = amount of biosorbent (g)

### 4.1 Parameters of biosorption

The biosorption process comprises the following steps: physico-chemical adherence, electrostatic bonding, ion-exchange, complexation, chelation, and precipitation.

#### 4.1.1 pH of the solution

The degree of antibiotic speciation and ionization, as well as the interactions between the biosorbent and the biosorbate, are significantly influenced by the pH of the solution and the surface charge of the algae cells. Tetracycline zwitterions are formed more easily in cultures with pH values between 7.5 and 8, which helps the drug adsorb onto algal surfaces [120]. At a pH of 6.0, arrhizus, activated sludge, and activated carbon were found to be present at 89.0, 66.0, and 61.0 mg/g. Changes in charge and surface characteristics impact on absorption at higher pH levels [107]. The biosorption percentage was found to increase along with the pH of the solution from pH 2.0 to 6.0. The degree of ionization of phenolic hydroxyls of tannin were also shown to increase with increasing pH value [121].

#### 4.1.2 Temperature

The enzyme activity and metabolic processes of biosorbents are influenced by temperature. Although higher temperatures generally improve reaction kinetics, they can adversely affect the performance of biosorbents. Extreme temperatures have the potential to disrupt kinetic rates [50]. For example, Rico et al. discovered that 30°C and 20°C were the ideal temperatures for algal cells, such as *Scenedesmusobliquus*, to biosorb. According to another study, dsorption capacity increased as the temperature rose from 278.15 K to 303.15 K and then steadily dropped to 323.15 K [120]. This phenomenon was shown to potentially be a result of the weakening of bonds between dicloxacillin and the active sites of the adsorbent at high temperatures [121].

#### 4.1.3 Contact time

Contact time refers to the time required for the binding of an antibiotic pollutant onto the surface of a biosorbent. To establish equilibrium, kinetic studies are essential to evaluate the performance of a biosorbent in removing antibiotic residues. In general, biosorption efficiency increases with increasing contact time. Rapid antibiotic biosorption by a biosorbent is needed to provide a shorter contact time between the biosorbent and a contaminant.

It has been reported that *Chlamydomonas reinhardtii* and *Dunaliella tertiolecta* were able to remediate 87% and 76% of fluoroquinolones and macrolides, respectively, within 1 hour of contact time because of the high growth rate of these microalgal species [122]. Another study reported the 95% and 94% biosorption efficiency of *Cladophora* sp. and *Spirulina* sp., respectively, for the remediation of tetracycline from aqueous solution within 2.5 hours of contact time (Abd and Ridha 2021). *Nerium oleander*-mediated silicon nanoparticles showed 98.62% biosorption efficiency for tetracycline biosorption from wastewater within 40 minutes [123].

#### 4.1.4 Biosorption kinetics and isotherm models

The kinetics of biosorption are directly related to the surface area of the biosorbent. Thus, biosorbent size is one of the predominant factors that affects biosorption efficacy. Another critical parameter in this process is ionic strength, which influences the biosorption of pollutants onto the surface of a biosorbent. The ionic strength is based on a broad range of electrostatic interactions in this process. Isotherm models, such as the Langmuir, Freundlich, and Temkin models, provide information about the surface properties of biosorbents and pollutants, as well as the affinity of binding sites of biosorbents and the uptake mechanisms. The Langmuir isotherm model implies that the uptake mechanism occurs on a homogeneous surface by monolayer biosorption with no interaction between biosorbed molecules. Whereas, the Freundlich isotherm model suggests that the uptake mechanism occurs on a heterogeneous surface by bilayer biosorption. It has been reported that for efficient biosorption of tetracycline from aqueous solution by *Cladophora* sp. and *Spirulina* sp., the biosorption process best fitted the Langmuir isotherm model [124]. By contrast, *Nerium oleander*-mediated silicon nanoparticles showed 98.62% biosorption efficiency for tetracycline by following the Langmuir isotherm model, suggesting that the biosorbent has a homogeneous monolayer surface [123].

### 4.2 Mechanism of biosorption

**Fig. 2:**
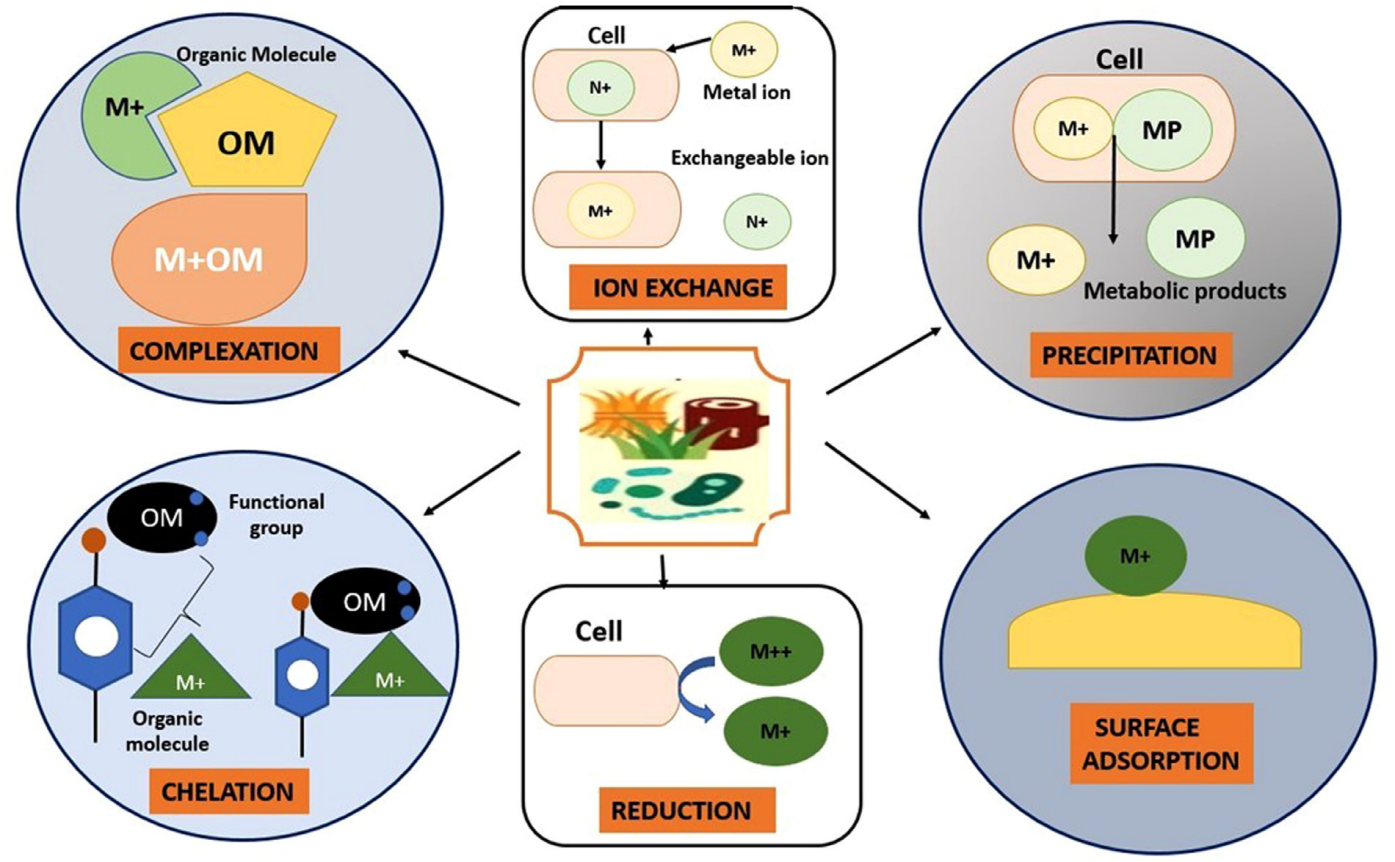
Different mechanism of Biosorption technique.

The biosorption of antibiotic residues is mainly carried out in batch experiments. The adsorption process was generally considered to be an exothermic process, but recently, it has been reported that the biosorption process can be either exothermic or endothermic in nature [125,126]. There are two types of biosorption, namely physisorption (physical biosorption) and chemisorption (chemical biosorption). When biosorbents such as microalgae, microalgae, fungi, bacteria, and medicinal plants are exposed to a solution containing antibiotics (i.e., a sewage water sample), these organisms show a series of responses to survive, one of which is degrading the harmful residues of antibiotics [93]. During the degradation process, biosorption takes place, which has been considered the most reliable method among the available degradation methods. The biosorption mechanism depends on four main pathways, namely ion exchange, pi-pi bond interactions, functional group and H-bond interactions, electrostatic interactions, pour filling, and intra-particle diffusion. In the ion exchange mechanism, the electrical neutrality of the aqueous solution is maintained [21]. This exchange mechanism using modified char has in some studies been found to be involved in the biosorption of tetracycline and ciprofloxacin. Another important pathway or mechanism involved in this process is intra-particle diffusion and pour filling with the help of biochar, which is positively correlated with the quantity of biosorption [117]. This mechanism mainly involves surface biosorption, in which specific antibiotic residues are directly adsorbed onto the surface of the biosorbent after ion exchange and internal diffusion of the particles. Electrostatic interactions, H-bonds, pour filling, and hydrophobic interactions are the main mechanisms for antibiotic biosorption on carbon-based biosorbents [82]. Antibiotic residues are adsorbed onto a specific biosorption surface through a physical attachment or interaction. Interactive biosorption is the most common biosorption pathway for antibiotics because it has multiple active sites in the form of functional groups, such as -COOH, -OH, -NH_2_, -CHO, C=O, and =SO_2_, and electrostatic points containing heterogeneous atoms [84,127]. The interaction between the functional groups of the biosorbent and the antibiotic residues can be defined as interactive biosorption. For example, -COOH groups represent more affinity towards polar silanol groups [97]. C=O groups show a higher affinity for the -OH group through cationic or H-bonding. Many adaptations have been found to improve the polar structure and surface properties of bacteria (e.g*., Pseudomonas putida*), plants (e.g., *Moringa oleifera*), fungi (e.g., *Saccharomyces cerevisiae*), algae (e.g., *Cladophora hutchinsae*), and agricultural waste products, such as rice husks, rice straws, and fruit peels. Tetracycline is the most studied antibiotic in terms of the biosorption of antibiotic residues because it is a common, broad-spectrum antibiotic. Tetracycline contains polar functional groups, namely carboxyl and acylamino groups. Ciprofloxacin also contains non-polar functional groups [127]. infrared spectroscopy (FTIR), X-ray powder diffraction analysis (XRD), and scanning electron microscopy (SEM) analysis of the biosorbents are carried out before and after biosorption of specific antibiotic residues [127]. Using XRD apparatus, different peaks are observed that correlate with different entities. For example, in the case of tetracycline (TC) adsorption with pumice stone, the main peaks of pumice stone and TC were at wavenumbers of 800, 1700, and 3500 cm^‒1^. SEM images of biosorbents are related to their surface properties, such as pore size, diameter, total volume, and external surface area. The biosorption process is highly dependent on pH. Different biosorbents have different adsorption capacities for antibiotic residues at different pH values. The biosorption capacity (*Q*_e_) and biosorption partition coefficient (KD) values are proportional to each other. Their values are inversely proportional to the pH value of the solution (i.e., the sewage water sample). The surface of the biosorbent has different ionic charges according to different pH values, and thus the bonds between the antibiotic and the biosorbents are formed depending on the pH of the solution. Therefore, biosorption experiments are carried out at the optimal pH of the solution. Another important parameter is the effect of the adsorption dose on the biosorption process. According to dose-dependent calculations, the percentage removal of antibiotic residues increases with increasing biosorption dose over a certain area. The increase in the biosorption of antibiotics is due to the presence of a greater number of active binding sites and a larger biosorption surface [114] (Fig. 2).

### 4.3 Different classes of antibiotic residues and their biosorption

Various types of biosorbents are investigated and studied for the efficient remediation of detrimental antibiotic residues from the three major types of wastewater mentioned in this review. Below the antibiotics that are found to be present in higher concentration in sewage water than other antibiotics are described (Table 2).

**Table 2:** Biosorption efficiency (*Q*_max_) of different biosorbents for different classes of antibiotics from waste effluents.

Ciprofloxacin	Banana stalk extract	*Q*_max_ = 49.7 mg/g Removal efficiency = 95% Surface adsorption, Electrostatic attraction Time = 30 min Temp.= 323 K pH = 4.5
Meropenem	Rice husk extract	*Q*_max_= 43.5 mg/g Removal efficiency = 95% Hydrogen bond and surface complexation Time = 3 h Temp. = 25–35˚C pH = 7.0
Sulfonamide	Artemia species extract	*Q*_max_ = 90 mg/g Removal efficiency = 98.06% Pi bond and electrostatic interaction Time = 5 h Temp. = 25˚C pH = 5.0–8.0
Ceftazidime	Chlorella pyrenoidosa surface	*Q*_max_ = 92.70 mg/g Removal efficiency = 96.07% Hydrophobic interaction and Vanderwaal formation Time = 24 h Temp. = 25˚C pH = 7.5
Metronidazole	Chlorella vulgaris surface	*Q*_max_ = 151.6 mg/g Removal efficiency = 50.08%–89.08% Hydrogen bond, pi-pi dispersion bond, CH-pi bond Time = 25 min Temp. = 25–35˚C pH = 5.0–7.5
Cephalosporin	Surface of *Chlorella* sp. Cha-01(ergosterol)	*Q*_max_ = 4.74 mg/g Removal efficiency = 67.82% Complexation and Chelation Time = 36 h Temp. = 26˚C pH = 7.5

#### 4.3.1 Tetracycline

Tetracycline is a wide-spectrum, bacteriostatic antibiotic that is prescribed to treat infections caused by both Gram-positive and Gram-negative bacteria. The bacteria *Pseudomonas pseudomallei, Campylobacter* sp., *Helicobacter* sp., *Brucella* sp., *Xanthomonasmaltophilia, Neisseria gonorrhoeae, Bacillus anthracis, Escherichia coli, Listeria monocytogenes*, and *Streptococcus pneumoniae* are sensitive to tetracycline. This antibiotic is frequently detected in almost all water systems including waste, drinking, and fresh water systems. The tetracycline molecule is strongly charged due to the presence of phenolic diketone, tricarbonylamide, and dimethylamine groups [127].

In one study, pumice stone was used as a biosorbent and the mechanisms involved surface complexation and cation exchange pathways. Maximum biosorption capacity was found to be 37.09 mg/g at a pH of 3 [128]. Another study showed that water hyacinth roots conferred 58.9%–84.6% removal at an optimum pH range of 4 to 6 [129]. It has been reported that ceramsite substrate is an excellent biosorbent for the removal of tetracycline residues from wastewater systems, and is extracted from bentonite, red mud, and pine sawdust. The maximum biosorption capacity of this substrate was 2.13 mg/g. Electrostatic interactions, hydrophobic interactions, and hydrogen-bonding were the main biosorption mechanisms in this study [130]. A study reported that the biosorbents extracted from shrimp shell waste (SSW) had a maximum biosorption capacity of 229.98 mg/g for 36 hours at a temperature of 55°C [117]. SSW is a mesoporous material with a reported average pore diameter of 4.47 nm and the presence of C-H and C=O groups on the cell surface. Here, both hydrogen bonds and pi-bonds formed between the target antibiotic and the SSW biosorbent at a low pH value of 3.3 [117]. Another study showed the practical application of iron (III)-loaded cellulose nanofibers as a biosorbent for the remediation of tetracycline, with a maximum adsorption capacity of 294.12 mg/g at pH 7. In this case, surface complexation was the dominant pathway and hydrogen-bonding, electrostatic interactions, and van der Waals forces were the three major interactions involved between the biosorbate and biosorbent [131].

#### 4.3.2 Dicloxacillin

This antibiotic is widely used both in human and animal livestock production and is considered a wonder antibiotic because of its first accidental discovery by renowned scientist Alexander Fleming. This antibiotic is used for the treatment of severe bacterial infections caused by *Staphylococcus* spp*.* and *Streptococcus* spp*.* [121], and is still widely used despite the development of resistance. Dicloxacillin is a beta-lactam antibiotic that belongs to the same class as penicillin.

Treatment of wastewater is an important step before it is released into fresh water systems, such as rivers and lakes. Tannin is a suitable, low-cost biosorbent employed for the biosorption of certain antibiotics. Tannin belongs to a class of plant secondary metabolites, which are water-soluble polyphenolic compounds. It has a molecular weight of >500 Da and is isolated from *Terminalia catappa L*. leaves [132]. A study focused on the remediation of antibiotic residues from pharmaceutical wastewater revealed that this biosorbent had a maximum capacity of 17.28 mg/g biosorption at a pH of 6.0 within 24 hours of treatment. Furthermore, dicloxacillin and the tannin were shown to acquire hydrogen bonds and van der Waals forces. The functional groups of tannin, including hydroxyl groups and carbonyl groups, reacted with this antibiotic via intermolecular bonds [121,133]. Researchers have only conducted one investigation on the biosorption of dicloxacillin to date. Therefore, more research on the removal of dicloxacillin using different biosorbents is necessary.

#### 4.3.3 Ciprofloxacin

Ciprofloxacin is an antibiotic belonging to the subclass of fluoroquinolones, used primarily for the treatment of urinary tract infections, sexually transmitted diseases, and skin and bone infections [134]. It inhibits bacterial DNA replication by inhibiting DNA topoisomerase and DNA gyrase. It has also been shown to be active against *Pseudomonas aeruginosa* [135,136]. Residue of this antibiotic in semi-digested metabolic products is widely discharged, with an abundance of up to 84% in wastewater [134].

Many studies have been conducted on the remediation of these residues using different biological biosorbents. Banana stalk-generated activated carbon, which is an agricultural waste, as well as an environmental-friendly biosorbent material, was investigated for its potential in ciprofloxacin remediation from wastewater. The value of monolayer biosorption capacity was 49.7 mg/g at a pH value of 4.5 and a temperature of 323 K [137]. This was a physical biosorption mechanism. Another research study reported that single-walled carbon nanotube biosorbed ciprofloxacin by hydrogen-bonding interactions and pi-pi interactions, and biosorbed around 99% of ciprofloxacin from the pharmaceutical and hospital wastewater [138]. One research study reported that thermally-modified bentonite clay material showed a larger surface area and a greater number of functional groups and ionic charges. Silica and amine groups were present on the surface of the given biosorbents, which favoured the biosorption mechanism due to electrostatic interactions between the biosorbate and biosorbents. Overall, 95% of biosorbate was removed from the wastewater in this study [114].

#### 4.3.4 Meropenem

Meropenem is a new, broad-spectrum antibiotic, belonging to the carbapenem class, that is used to treat severe skin or stomach infections, as well as bacterial meningitis, pneumonia, sepsis, and intra-abdominal infection [139]. The overuse of meropenem has led to widespread resistance among almost all bacteria. Residues of this antibiotic are difficult to remove from water treatment plants and thus end up in rivers, lakes, seas, and finally drinking water and food [140,141]. Research is lacking on the removal of meropenem residues from water systems using adsorption technology. However, such research is vital as meropenem is the last line of defence against serious bacterial infections. One report showed that a biosorbent functionalized with Mg/Fe layered double hydroxides from rice husk had effective biosorption capacity (up to 43.5 mg/g) toward the target antibiotic. The biosorption process, which was conducted at 250–350°C, predominantly involved the formation of surface complexes and physisorption [142]. Another study showed that the removal efficiency using biosorbable lignocellulose from sawdust waste was 92% for the target antibiotic, and the removal efficiency of the post-treated biosorbent was 96% [143].

#### 4.3.5 Ceftazidime

Ceftazidime is a semi-synthetic drug, classified as a third-generation cephalosporin, which has broad-spectrum activity. It is particularly active against *Enterobacteriaceae*and *P. aeruginosa*. It is also widely used to treat lower respiratory tract infections, and complicated or chronic urinary tract infections. It functions as a beta-lactamase inhibitor. Excessive production and use of ceftazidime and other similar antibiotics has contributed to the release of harmful residues into aquatic environments. Since 2001, several widely used antibiotics, such as ceftazidime, sulfonamide, meropenem, cephalosporin 7-ACA, and tetracyclines, have been found to be present in pharmaceutical, hospital, and domestic wastewater as a result of the higher levels of antibiotic residues in animals and humans [144]. One study showed that the ceftazidime-tolerant green alga *Chlorella pyrenoidosa*is an effective and suitable biosorbent for the removal of the target antibiotic from wastewater and showed a maximum biosorption capacity of 98.34%. Functional groups, such as amino, hydroxyl, and carboxyl groups, on the surface of certain biosorbents were involved in the biosorption mechanism. The dead algal cells conferred a maximum removal efficiency of 99.20%. This process involved electrostatic interactions and hydrogen bonding [145]. Another study indicated that carbon nanomaterials, such as carbon nanotubes, are excellent organic pollutant biosorbents with a large surface area, high biosorption capacity, and rapid treatment time. In this study, multi-walled carbon nanotubes were used and the dose ranged from 0.02 g to 0.20 g, while the ceftazidime concentration was 30 mg/L, the pH range was 4.0 to 11, and the contact time was 90 minutes. In addition, functional -OH groups competed for the biosorption site with negatively charged ceftazidime, with a removal efficiency of 80%–90% and a biosorption capacity of 15.24 mg/g [130]. Another study reported that coal fly ash-derived zeolites had a maximum biosorption capacity of 80 mg/g during 20 minutes of stirring at a temperature range of 45–950°C.

#### 4.3.6 Sulfonamides

Sulfonamide antibiotics competitively inhibit PABA incorporation into folic acid (folic acid is essential for the proliferation or growth of bacteria), inhibiting the synthesis of folic acid [146]. These antibiotics are synthetic antibacterial compounds, with broad-spectrum activity against both Gram-positive and Gram-negative bacteria. In recent years, several studies have shown that most bacteria have developed resistance to sulfonamides [147]. Sulfonamide resistance is linked to an amino acid substitution in the dihydropteroate synthase (DHPS) enzyme, which prevents binding of this antibiotic. The general MRL established in European Union (EU) regulation no. 37/2010 for sulfonamides specified that the concentration in meat matrices should not exceed 100 µg/kg [148]. However, the MRL value has been exceeded in water and food products, and as a result bacteria such as *Streptococcus* sp., *Proteus* sp., *E. coli, Pasteurella* sp., *Nocardia* sp., *Klebsiella* sp., *Pseudomonas* spp., and *Enterococci* sp., have become resistant to sulfonamides, with potential adverse effects on human and animal health. Harmful sulfonamide residues remain in wastewater plants permanently, and even at low concentrations can endanger the health of humans, animals, and plants. Thus, the thorough removal of sulfonamide residues from wastewater is urgently needed [146].

One study reported that chitosan is the most reliable biosorbent for the remediation of sulfonamides in a wide range of wastewater because of its stability at high temperature and high pH [149]. Graphene oxide (GO) can also be used as an biosorbent as it enhances the dispersitivity level of composites, has a large surface area, and has a large number of functional groups that include =O and -COOH present on the edges and -OH on the surface [147]. Sulfonated graphene oxide, which has a negatively-charged surface, therefore showed high affinity between its sulfo group and sulfonamide residue pollutants [150]. Carboxymethyl cellulose (CMC) was also shown to be a suitable biosorbent, possessing carboxymethyl and hydroxyl groups, and CMC/SGO-GCC showed a maximum biosorption capacity of 87% at a pH of 6 or 8 within 30 minutes [151]. Another study revealed that the three antibiotic residues of sulfadiazine, sulfamethazine, and sulfachloropyridazine were remedied using three different biosorbent materials, pine bark, oak ash, and mussel shell. Pine bark demonstrated higher affinity for these three antibiotic residues than the other two biosorbents. Pine bark absorbed up to 95% of the given antibiotics within 24 hours of contact time [149]. According to another study, carbonaceous materials with a pH of 4.0, such as powdered activated carbon, granular activated carbon made of wood, and graphene, showed 90%–95% of their biosorption capability after only 5 hours of contact time. At a temperature of 250°C and a pH range of 5.0–8.0, sulfonamides adsorbed onto the surface of the diatom *Chaeto ceros* and the arthropod *Artemia* within 24 hours and 5 hours of contact time, respectively, with biosorption capabilities of 88% and 90%, respectively. According to another research study, MIL-53s, which are porous metal-organic frameworks with ligands and metal clusters, can be employed for drug administration, gas storage, catalysis, adsorption, water purification, and pore-size adjustment, and MIL-53 (Cr), MIL-53 (Al), and MIL-53 (Fe) were tested as biosorbents [151,152]. The target contaminant concentration was 20 mg/L, the biosorbent dosage was 5 g each, the temperature was 298 K, the duration was 24 hours, and the pH range was 3–6 [152]. The maximum biosorption capacities of the biosorbents were 0.348, 0.349, and 0.0369 mmol/g, respectively, while sulfonamide was biosorbed onto the MIL-53s via pi-pi interactions, hydrophobic interactions, van der Waals forces, and electrostatic interactions [1]. According to another study, biochar and hydrochar, generated from used coffee grounds, showed 121.5 g/g and 130.1 g/g at 250°C, respectively, whereas biochar and hydrochar showed biosorption capacities of 82.2 g/g and 85.7 g/g at the same temperature, with pi-pi electron donor-acceptor interactions playing a role [126]. According to another study, carboxyl-functionalized biochar made from walnut shells had a clearance effectiveness of 99% for sulfonamide, via a mechanism involving hydrogen bonds and pi-pi interactions [153].

#### 4.3.7 Nitroimidazole

According to Sun et al. (2019), nitroimidazole antibiotics are frequently used to treat and prevent infectious diseases caused by anaerobic bacteria and protozoa. Investigations have revealed that these antibiotics are often present in industrial effluents, drinking water, fish farm water, and wastewater treatment plants [154]. Additionally, these antibiotics are potentially mutagenic and carcinogenic due to their high polarity, which makes them difficult to degrade [155]. Numerous studies have demonstrated that waste biomass biosorbents, rather than powdered biochar, are more suited, and easier to recover and regenerate in nature for the purpose of practical separation [156]. Carbon foam is a porous carbon material that is typically made from coal, coal tar pitch, and petroleum pitch. It is lightweight, has a wide surface area, and an open cell structure [84]. To remediate metronidazole (MNZ) and dimetridazole (DMZ), *Vallisneria natans*was employed as a waste biomass to create biomass carbon foam pellets, and it demonstrated biosorption capability of 64.23 mg/g in less than 90 minutes. As a result of their larger surface area, basic surface, and pi-pi stacking, single-walled carbon nanotubes demonstrated the highest biosorption capacity for MNZ and DMZ, according to another study. At a low pH of 2, the basic groups and the electrostatic interactions contributed to this biosorption mechanism. The Sheindorf–Rebuhn–Sheintuch biosorption model was employed in this context [155]. The biosorption of nitroimidazole onto activated carbon was the subject of another research investigation using microorganisms which revealed a maximum biosorption capacity of 2.04 mmol/g [116]. However, the microorganisms used in the biological stage of the wastewater treatment did not breakdown nitroimidazoles, but the number of microorganisms adsorbed onto the activated carbon during the adsorption process increased. As a result of interactions akin to pi-pi dispersion between carbon graphene layers and nitroimidazole aromatic rings, which were present in both the adsorbent and the biosorbate, electron-activating groups were able to initiate the adsorption process, and pH had little to no influence [116].

#### 4.3.8 Cephalosporin

Cephalosporins are beta-lactam antibiotics that disrupt the manufacture of peptidoglycan in Gram-positive and Gram-negative bacterial cell walls and are frequently used to treat and prevent serious bacterial illnesses. Wastewater containing cephalosporins poses a threat to the environment since it may contain a variety of organic chemicals, antibiotic residues, and various inorganic salts [157,158]. Antibiotic residues in aquatic environments have harmful effects, ultimately impacting the evolution of bacterial community structure and leading to the development of antibiotic resistance. One type of intermediate cephalosporin is 7-amino cephalosporanic acid (7-ACA), and the beta-lactam ring structure of this residue is responsible for its antibacterial activity. These residues are also known as semi-synthetic cephalosporins [158].

In a study three microalgal strains of *Chlorella* sp., *Chlamydomonas* sp., and *Mychonastes* sp. isolated from southern Taiwan had biosorption capacities of 4.74, 3.09, and 2.95 mg/g, respectively, at a pH of 7.5 and a temperature of 260°C. Their unique cell sizes, surface areas, and surface properties are responsible for their different biosorption capacities [159,160]. The Langmuir and Freundlich isotherm models were used to describe the mechanism of biosorption by these three types of microalgae biomass, which involved monolayer and multilayer biosorption onto the heterogeneous surfaces of these three microalgae [130]. Activated carbon was the first adsorbent utilized; however, issues with regeneration have hindered its application [159,160].

Another research study showed that cephalosporin 7-ACA, at a pH range of 4–8 and 10 hours of contact, conferred 89%–96% biosorption capacity. In this example, the surface structure of the activated carbon was mostly acidic polar carbon-oxygen, carboxylate, or lactonic groups, and hydrophobic interactions were mainly responsible for the high biosorption affinity at low pH [161].

## 5 Significance and advantages of the biosorption process for antibiotic residue remediation

The biosorption process offers unique advantages for removing antibiotic residues from wastewater. Tetracycline antibiotics, and their residues, are the well-studied of all antibiotics in terms of biosorption technology. Almost all biosorbents tested respond well to tetracycline antibiotics. Biosorption using biosorbents is cost-effective and environmentally friendly and has been proven to be the most effective and efficient process for cleaning antibiotic residues from wastewater [119,124,133,135]. Biosorbents including banana peel, *M. oleifera, P. putida, S. cerevisiae*, and other agricultural wastes, have been proven to be suitable for use in the biosorption of antibiotic residues from wastewater. Biosorbents can also be used in dry form, which means that it is not necessary to add nutritional supplements [93,126,162]. More research is needed to find more benefits of using this method to remove antibiotic residues in wastewater. Conventional technologies for the removal of wastewater pollutants have advantages and disadvantages. Chemical precipitation, dielectric barriers, and electrochemical coagulation treatment technologies are not always effective, especially when antibiotic residual pollutants in wastewater systems are present in low concentrations [163]. Furthermore, such treatment technologies release trace amounts of sludge that are difficult to remove. Other technologies, such as ion-exchange and chlorination, are complex and expensive [164]. To overcome such limitations, biosorption is a preferable wastewater treatment technology for antibiotic remediation. This process can easily remove large amounts of sludge with great ease without releasing any secondary pollutants. Moreover, this process is simple and feasible because there is no requirement for nutrients or energy [118,162]. Furthermore, electric coagulation is time-consuming and unable to completely remove antibiotic pollutants from wastewater. By contrast, the biosorbents used in the biosorption process can completely remediate emerging pollutants over a short timescale and can be applied to all types of wastewater system. Furthermore, aseptic conditions are not essential and since it is a metabolism-independent technology, it is even able to mitigate low concentrations of antibiotic contaminants from wastewater systems.

## 6 Future prospects

An “emerging pollutant” is a pharmaceutical molecule, such as an antibiotic residue, that has been newly detected in an aquatic environment, particularly in wastewater. For the elimination of antibiotic residues, a number of treatment techniques, including activated sludge and biodegradation, have been examined and studied. Following a physically regulated mechanism or an interactive mechanism, antibiotic residues are directly biosorbed onto a particular biosorbent surface. The biosorption procedure is frequently regarded as a viable, efficient, and eco-friendly technique for removing such antibiotic residual contaminants from wastewater. The biosorbents can then be regenerated and reused. Several biosorbents have been developed and exploited, and those that have already shown efficient antibiotic residue removal capacity include shrimp shell waste and *P. putida*. These biosorbents may benefit from chemical modification to increase their surface area and consequently their biosorption efficiency in the future. Genetically-modified plants and microorganisms should also be investigated to enhance biosorption efficiency and treatment time. Further investigations are needed in this field of research to address the global problem of antibiotic resistance. Moreover, a few research studies have reported difficulties in separating supernatants while using dead/living microorganisms or other mixed biomass, as it is necessary to separate supernatants to obtain environmentally-friendly biosorbents [163]. Thus, there is a need to find suitable inert supports for the encapsulation of microbes or biomass. The surface areas of a variety of microorganisms, plants, and other human-originated biomaterials have the capability to be used as biosorbents; however, for more efficient biosorption of antibiotic pollutants from wastewater, nano-sized biosorbents would potentially offer a larger surface area to which traces of antibiotic pollutants could be easily biosorbed, although biogenic nano-sized biosorbents for antibiotic pollutant eradication from wastewater systems are yet to be reported. Microalgae-based biosorbents have attracted attention because of their low cost and ability for sequestration of carbon dioxide and wastewater purification [76]. However, there are some key challenges of using microalgal biomass, such as their lower capacity to remove antibiotics, their lower efficiency to remove antibiotic toxicity, their intermediate transformation of secondary products, their undefined antibiotic removal pathways, and their inability to mitigate the impacts of wastewater-borne bacterial species. These challenges hinder the large-scale usage of this biosorption technique. Thus, there is a need to carry out more investigations for developing efficient real-time feasible solutions, possibly including enhancing the active sites on microalgal species [165]. In the future, the antibiotic removal efficacy of reused and recycled biosorbents needs to be investigated to achieve the remediation of antibiotic residues at a feasible cost and over an acceptable time period. To date, chemical reagents have been used for the desorption of antibiotic contaminants from the surface of biosorbents, which have shown maximum blockage of the biosorbent surface, thereby decreasing the efficiency of reused and recycled biosorbents for antibiotic remediation. Therefore, there is a need to find eco-friendlier, less adherent desorption agents. In real wastewater, multiple types of antibiotic residues are present, and it is technically difficult to remediate multiple antibiotic residues using the same biosorbent simultaneously. Therefore, there is a need for in-depth studies on biosorbents that are able to remediate all antibiotics and other pollutants from wastewater at the same time. The scope of the biosorption process will expand in the future, along with a range of new biosorbents able to biosorb valuable and hazardous emerging pollutants. The use of nano-sized biomass as a biosorbent for antibiotic pollutants may generate revenue for industries presently disposing of waste biomass at a cost.

## 7 Conclusion

Because many researchers have noted high concentrations and accumulations of harmful antibiotic residues, this review article presents a wide range of biosorbents used for the biosorption of different types of antibiotic residues from three types of wastewater, namely pharmaceutical, hospital, and municipal wastewater. The effective clean-up of antibiotic residues from pharmaceutical and hospital wastewater using biosorption technology was recently reported in multiple publications. The mechanism of antibiotic resistance and its reservoirs have been comprehensively addressed in this paper. Each antibiotic residue has a maximum permissible level, referred to as the MRL, in food and drink. Wastewater treatment facilities are a hotspot for the occurrence of antibiotic residues and the spread of antibiotic resistance in recent years. The development and spread of antibiotic resistance in humans and animals may have been aided by the residual levels of antibiotics in food and water exceeding the MRL. Different types of antibiotic residues have been detected in different water sources. For example, in one report, the concentration of amoxicillin residue in hospital wastewater was 900 ng/L, whereas the concentration of tetracycline residue was 9.6 × 10^3^ ng/L. Through a physically-regulated mechanism or an interactive mechanism, antibiotic residues adsorb onto a particular biosorbent surface. The electrostatic points of antibiotic residues, which are dependent on heterogeneous elements, including -F and -Cl, are also implicated in their biosorption when using a variety of natural biosorbents. The most effective and widely used adsorbent for the adsorption of antibiotic residues is CAC. However, carbon nanotubes may be a less effective adsorbent for effectively removing antibiotic residues from the aforementioned wastewaters, and according to several reports, chemically-altered biosorbents were more effective at biosorbing antibiotic residues from pharmaceutical, medical, and municipal wastewaters. Biosorbents may be subjected to acid/base treatment, hydroxylation, and carboxylation for surface modification. The phenomenon of antibiotic residue biosorption is influenced by various factors. Depending on the ionic strength of the solution, antibiotics can be present in cationic, anionic, or neutral form and are made up of multifunctional groups. It may be necessary to do additional, in-depth research into how the presence of secondary solutes affects the biosorption process.

It has been demonstrated that SSW, without any modification, is a practical biosorbent for the removal of tetracycline from aqueous solution at a pH range of 6.0–8.0. According to reports, -OH groups were involved, which led to the establishment of hydrogen bonds. Three distinct biosorbents were employed for the biosorption of tetracycline from hospital wastewater, namely mussel shell, pine bark, and oak ash. All three of these biosorbents demonstrated good tetracycline residue biosorption capacities and possessed the carboxylic acid functional group, -COOH. Additional biosorbents should be considered in the future for the remediation of other antibiotic residues. To date, biosorbents that have been used against one or more antibiotics have been shown to possess similar adsorption and removal capabilities. The goal of this review was to provide information on the biosorbents that have recently been employed to remove antibiotic residues derived from various antibiotic classes. Biosorption is an environmentally beneficial technique, compared with conventional techniques, and is therefore the preferred method to treat wastewater from hospitals, pharmaceutical companies, and municipalities. Research is ongoing into how to more effectively adsorb antibiotic residues from different types of wastewater using genetically modified biosorbents. Such research is important to improve the surface characteristics of biosorbents to ensure the effective biosorption of antibiotic residues from wastewater. There are extensive economic, social, and environmental benefits to researching and developing new biosorbents, and this review may serve as a resource for future biosorption research and the ongoing management of water pollution.

## Funding

This research did not receive any specific grants from funding agencies in the public, commercial, or not-for-profit sectors.

## Author contributions

B.M, A.K., and S.B. conceived the review, and wrote the first draft. All authors edited the manuscript into its final form.

## Acknowledgment

The authors thank Techno India University, West Bengal, for support and encouragement during this study.

## Declaration of competing interest

The authors declare no conflicts of interest.

## Data available statement

No data, models, or code were generated or used during the study.

## Ethics statement

An ethical statement is not required as there were no human subjects involved in this study.

## Informed consent

Informed consent was waived for this study because no patients’ data were reported.
